# The utilization of oral feeding in pediatric pancreatitis: a randomized controlled study

**DOI:** 10.1016/j.jped.2026.101501

**Published:** 2026-02-06

**Authors:** Yan Han, Hong Zhao, Linchen Fu, Xinyi Jia, Xiao Du, Liqun Zhou, Jindan Yu, Jie Chen, Jingan Lou

**Affiliations:** Department of Gastroenterology, Children’s Hospital, Zhejiang University School of Medicine, National Clinical Research Center for Children and Adolescents' Health and Diseases, Hangzhou, China

**Keywords:** Pediatrics, Acute pancreatitis, Nutritional therapy, Oral feeding, Enteral nutrition

## Abstract

**Objective:**

There is increasing awareness of the benefits of early nutritional intervention in children with acute pancreatitis (AP). This study aims to compare oral feeding with NG feeding in mild to moderately severe AP patients.

**Methods:**

A single-center, prospective, randomized controlled trial was conducted from September 2021 to August 2024. The participants were randomly assigned to the oral feeding group or the NG feeding group. The primary outcomes were the duration of AP-related pain, tolerance rate, and changes in weight.

**Results:**

A total of 56 pediatric patients were enrolled, of whom 48 patients (24 in each group) were included in the final analysis. There were no significant differences in baseline characteristics or etiological analysis results between the two groups. The median duration of abdominal pain after admission was 3 days in both groups (*p* = 0.104); no difference was found in the tolerance rate between the 2 cohorts (*p* = 0.489). There were no significant differences in weight change between the two groups at discharge or at 1 week or 5 weeks after discharge (*p* = 0.658, 0.502, and 0.927, respectively), and both groups presented slight increases in weight at 5 weeks post-discharge. Four patients in the NG group developed complications, while no complications were observed in the ORAL group (*p* = 0.109).

**Conclusions:**

Oral feeding is effective for nutritional therapy in children with mild to moderately severe AP, reducing the number of invasive procedures, without significant adverse effects.

## Introduction

Acute pancreatitis (AP) refers to the activation of pancreatic exocrine enzymes due to various etiologies [1,2]. Its diagnosis in children requires at least two of the following: abdominal pain consistent with AP, serum amylase and/or lipase levels ≥ 3 times the upper limit of normal, or imaging findings indicative of AP.(2) As a common acute abdominal condition, AP is clinically categorized by severity into mild, moderately severe, and severe forms [3]. Mild AP involves no organ failure or complications, and typically resolves within one week; moderately severe AP (SAP) is defined by either transient organ failure or dysfunction (resolving within 48 h) or the presence of local or systemic complications; SAP refers to pancreatitis with persistence of organ failure lasting beyond 48 h [3]. In recent decades, there has been a notable increase in the incidence of AP among pediatric populations [4,5].

Enteral nutrition (EN) is the preferred method of clinical nutritional support for children and is indicated for the management of conditions such as pediatric AP and pediatric Crohn's disease [2,6]. EN offers clear benefits for AP patients, shorter hospital stays, lower intensive care unit (ICU) admission, and reduced SAP incidence versus parenteral nutrition [7,8]. Inappropriate nutritional interventions may compromise children's immune response and exacerbate complications, thereby impeding recovery. Therefore, judicious nutritional support is paramount for managing pediatric AP.

Several guidelines recommend early EN as the primary therapy for pediatric AP, suggesting oral feeding over nasogastric (NG) feeding in mild-to-moderate cases [2]. However, these are predominantly based on evidence from adults and limited pediatric research [8,9]. Our previous randomized controlled trial confirmed that NG feeding is safe and effective in pediatric AP;[10] whereas in adults with high-risk AP, early nasoenteric feeding was not superior to an oral diet initiated after 72 h [11]. This prospective study compared oral and NG feeding, based on the hypothesis that oral feeding would be as safe and effective as or non-inferior to NG feeding.

## Methods

A single-center, prospective, randomized trial was conducted at a tertiary center, the Children’s Hospital, Zhejiang University School of Medicine in China, from September 2021 to August 2024. The participants were patients who were diagnosed with AP and required hospitalization. The study was approved by the Ethics Committee of the Children’s Hospital, Zhejiang University School of Medicine, and was carried out in accordance with the Declaration of Helsinki. The purpose of the study was explained clearly and accurately to all patients and guardians, and their informed written consent was signed. The trial was registered at the Chinese Clinical Trial Registry (ChiCTR2100050915).

### Criteria

The inclusion criteria were as follows: ① age ranging from 2 to 18 years, irrespective of sex; ② diagnosis of AP or recurrent AP [12], graded as mild AP or moderately SAP, and treated in accordance with established protocols for AP; and ③ written informed consent obtained from parents/legal guardians.

The exclusion criteria were as follows: ①SAP; ② chronic pancreatitis and acute exacerbation of chronic pancreatitis; ③ the presence of absolute contraindications for EN (e.g., severe hemodynamic instability, intestinal ischemia/necrosis, or obstruction); ④ children who had already initiated nutritional therapy prior to enrollment; ⑤ the presence of underlying diseases (e.g., congenital heart disease, severe liver or gallbladder diseases, immunodeficiency, or cancer); ⑥ participation in other clinical trials within the past six months; ⑦ situations deemed unsuitable for inclusion by the researchers (e.g., undiagnosed underlying conditions, geographical inaccessibility for follow-up).

### Study design

All patients were randomly assigned in a 1:1 ratio to two groups: the oral feeding group (referred to as the ORAL group) and the nasogastric feeding group (referred to as the NG group). Randomized sequences were generated via SAS 9.0 (SAS Institute, Cary, NC). The study commenced immediately following randomization. Blinding in the trial was not feasible because of the natural characteristics of the nutritional methods used. In the NG group, patients received EN therapy through a Flocare® NG tube (Nutricia, Wuxi, China), which was positioned at the bedside in the ward and confirmed by aspiration or radiography.

All participants initiated EN within 48 h after admission. The individualized estimated energy requirement (EER) was standardized through calculations and confirmations by clinical nutritionists. The maximum EER did not exceed 2000 kilocalories per day [10]. To minimize variability, a polymeric formula (463.7 kilocalories per 100 *g*, Supplementary Table 1) served as the exclusive energy source for both groups prior to transition to a full-fat oral diet. The nutritional target for both groups was to achieve > 70 % of the EER within 3 days, and then maintained for 2 subsequent days. In the NG group, feeding was advanced according to a stepwise protocol based on our experience and adult trial: [10,13] one‑third of the EER was delivered on the first day, followed by a one‑third increment each day until the target dose was reached on day 3. If there was an improvement in pain, accompanied by a decrease in serum amylase and/or lipase levels or the attainment of acceptable standards as determined by the attending physician, the patients might transition to a full-fat oral diet. Prior to resuming the oral diet, the NG tube was removed.

Patients in both groups received standard treatment according to their clinical needs, which encompassed fluid therapy and antibiotic administration. Comprehensive baseline characteristics and clinical data were documented, encompassing hematological analysis, electrolyte levels, blood glucose, renal and hepatic function, serum amylase and lipase levels, and arterial blood gas analysis. Contrast-enhanced abdominal computed tomography was used to evaluate initial pancreatitis severity. Magnetic resonance cholangiopancreatography (MRCP) was used to delineate the biliary tract and pancreatic ducts. Transabdominal ultrasound (US), known as non-invasive and radiation-free, was used to monitor disease progression and recovery at baseline and follow-up visits.

The recurrent pain associated with AP is characterized by the reappearance of abdominal discomfort and is accompanied by a twofold or greater increase in serum amylase and/or lipase levels than previously reported. Patients experiencing recurrent abdominal pain should reduce their food intake to alleviate symptoms. If the reduction is effective, they might gradually increase their degree of feeding as necessary; if not, they should continue to reduce it until they fast. Intolerance was defined by the recurrence of symptoms (e.g., abdominal pain, nausea, vomiting, fever, or diarrhea) after initial relief during EN, which persisted despite a reduction in feeding rate and ultimately necessitated suspension of EN and a modification in the feeding regimen. Intolerant patients fasted and received intravenous nutrition prior to any changes in feeding methods. For children who underwent adjusted feeding, upon reaching 75 %−100 % of the EER, the nutritional objective was achieved, paving the way for subsequent planning. If a patient in the oral feeding group was unable or considered unlikely to start oral feeding within 48 h of an attack, nasogastric or nasojejunal tube insertion for EN was considered.

After 1–2 days of an oral full-fat diet, the patients were discharged. However, the authors continued to monitor them at two scheduled follow-up time points: one at 1 week (7 days) post-discharge and the other at 5 weeks (35 days) post-discharge. At these two time points, the patient was required to report clinical symptoms and undergo weight measurements, blood tests, and ultrasound examinations.

The primary outcomes were the duration of AP-related pain, the feeding tolerance rate, and changes in weight. The secondary outcomes included recurrent abdominal pain, clinical nutrition-related laboratory parameters, alterations in amylase and lipase levels, ultrasonographic changes, readmission of recurrent pancreatitis, local and systemic complications of AP, and complications.

### Sample size

Published studies have suggested that the length of hospital stay was 9 (5–12) days in the NG group[14] and 2.6 (2–4) days in the oral group [15]. At a significance level of 5 % (two-sided), an effect size of 90 %, and a dropout rate of 20 %, an estimated sample size of >34 per group is needed. Given the characteristics of the pediatric population, the necessity for guardian-informed consent, and the experience gained from the aforementioned study[10] the authors anticipate challenges in achieving the sample size; therefore, the authors endeavored to recruit as many eligible participants as possible during the experimental period.

### Statistical analysis

The statistical analysis was performed via SPSS 26.0 (SPSS Inc., Chicago, IL, USA). Categorical variables are presented as frequencies with percentages and were compared via the χ2 test or Fisher’s exact test. Continuous variables with a normal distribution are reported as the means and standard deviations and were analyzed via Student’s *t*-test. Continuous variables with a nonnormal distribution are reported as medians and interquartile ranges (IQRs) and were analyzed via the Wilcoxon rank test. A P value of <0.05 (two-tailed test) was regarded as statistically significant.

## Results

**Fig. 1:**
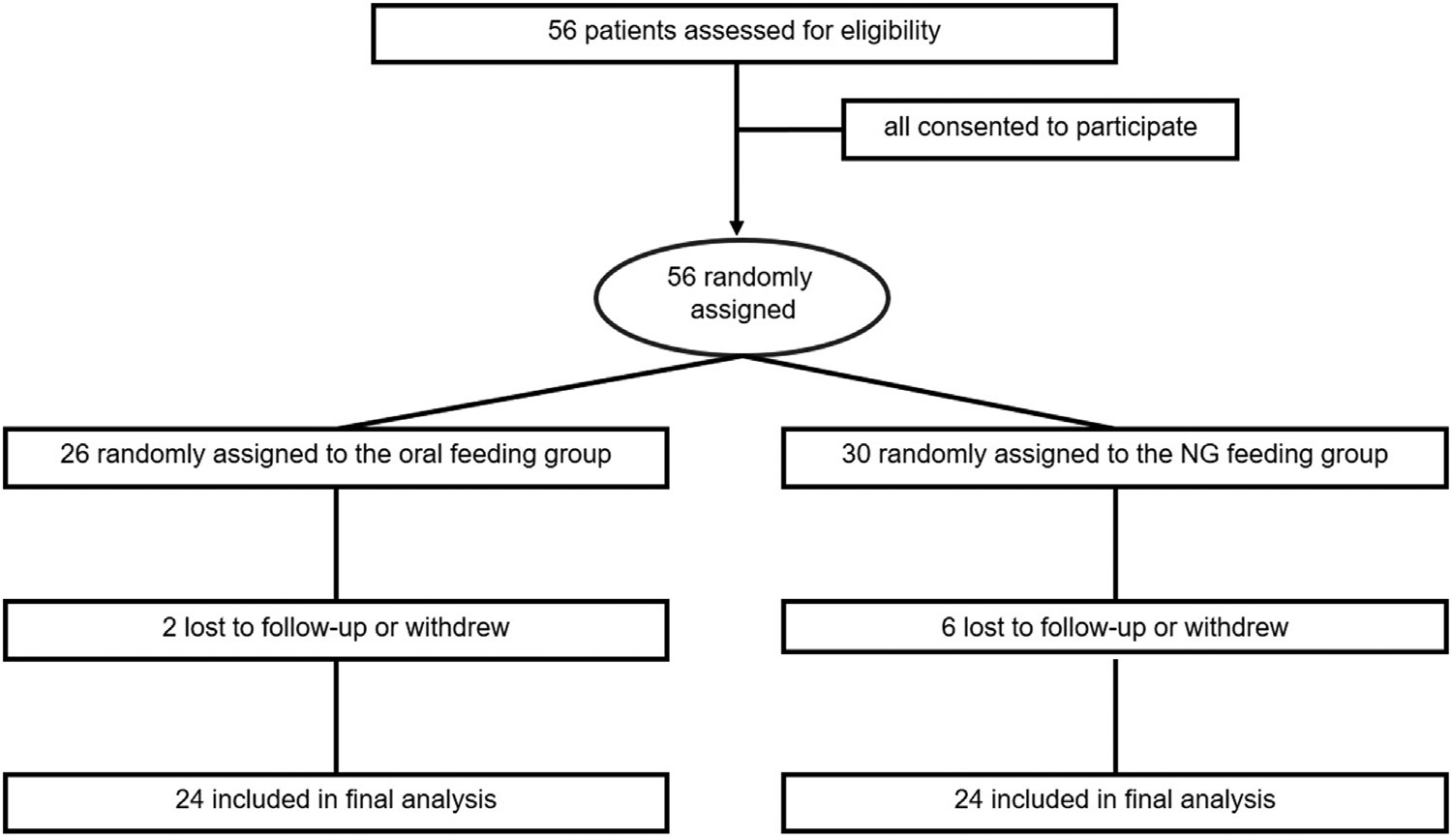
Recruitment, randomization, and follow-up in clinical trials.

Figure 1 shows the CONSORT flowchart of this study. A total of 56 patients were recruited, with 26 (46.4 %) participants randomly allocated to the ORAL group and 30 (53.5 %) to the NG group. Two patients from the ORAL group either withdrew from the study or were lost to follow-up, whereas 6 patients in the NG group experienced similar outcomes. Forty-eight participants successfully completed the protocol, with an equal distribution of 24 patients in each group. The ORAL group included 2 patients with moderately SAP and 22 with mild AP, compared to 4 and 20 patients, respectively, in the NG group (*p* = 0.382). There was no difference between the 2 groups in demographic and anthropometric characteristics or laboratory and imaging examinations at presentation (Table 1). With respect to clinical presentation, all patients experienced abdominal pain prior to admission, with an average duration of approximately 1.5 (0.6 to 3.8) days for the ORAL group and approximately 1.0 (0.5 to 3.8) days for the NG group (*p* = 0.975). The most prevalent accompanying symptom was vomiting, which occurred in 45.8 % of the ORAL group and 66.7 % of the NG group (*p* = 0.146, Table 2). In terms of etiology, anatomical abnormalities were observed as the primary cause, followed by idiopathic factors. There was no difference in etiology between the groups (Table 3).

**Table 1:** Baseline characteristics.

	ORAL group (n = 24)	NG group (n = 24)	P value
Age, y, median (IQR)	7.6 (4.8 to 9.8)	8.8 (5.0 to 11.4)	0.433
Gender (male/female), n	12/12	15/9	0.388
Height, cm, mean±SD	127.1 ± 18.4	132.2 ± 24.2	0.412
Weight, kg, median (IQR)	24.3 (18.1 to 38.6)	26.5 (17.4 to 45.3)	0.643
Amylase, U/L, median (IQR)	308.2 (100.8 to 1222.0)	556.7 (227.8 to 1398.0)	0.183
Lipase, U/L, median (IQR)	499.5 (215.0 to 934.8)	603.5 (312.3 to 1039.3)	0.509
WBC, *10^9/L, median (IQR)	8.7 (7.8 to 13.7)	13.2 (8.0 to 16.1)	0.106
Hb, g/L, median (IQR)	124.5 (117.3 to 130.0)	127.0 (120.3 to 135.0)	0.212
CRP, mg/L, median (IQR)	3.8 (1.4 to 23.6)	2.1 (0.5 to 9.3)	0.403
Albumin, g/L, median (IQR)	42.6 (39.0 to 45.6)	41.9 (40.1 to 44.5)	0.595
ALT, U/L, median (IQR)	14.0 (10.3 to 22.3)	17.0 (11.3 to 25.8)	0.495
AST, U/L, median (IQR)	30.5 (21.3 to 41.8)	26.0 (21.0 to 56.0)	0.662
PAB, mg/L, median (IQR)	174.8 (133.1 to 205.9)	182.8 (149.0 to 203.1)	0.444
Cr, μmol/L, median (IQR)	35.0 (26.3 to 39.0)	35.0 (28.3 to 37.8)	0.749
BUN, mmol/L, median (IQR)	4.3 (2.7 to 4.9)	3.4 (2.8 to 5.2)	0.813
LDH, U/L, median (IQR)	242.0 (211.3 to 404.0)	277.0 (210.0 to 372.0)	0.966
Ca, mmol/L, median (IQR)	2.4 (2.3 to 2.4)	2.4 (2.3 to 2.5)	0.632
P, mmol/L, median (IQR)	1.5 (1.4 to 1.7)	1.5 (1.3 to 1.6)	0.148
TG, mmol/L, median (IQR)	0.7 (0.5 to 0.9)	0.6 (0.5 to 1.0)	0.823
CHOL, mmol/L, median (IQR)	4.3 (3.7 to 4.7)	4.5 (3.5 to 5.1)	0.482
Imaging abnormalities, n ( %)	24 (100)	24 (100)	1.000

NG, nasogastric; WBC, white blood cell; Hb, hemoglobin; CRP, hypersensitive C-reactive protein; ALT, alanine aminotransferase; AST, aspartate aminotransferase; PAB, prealbumin; Cr, creatinine; BUN, blood urea nitrogen; LDH, lactic dehydrogenase; Ca, calcium; P, phosphorus; TG, triglycerides; CHOL, cholesterol; SD, standard deviation; IQR, interquartile range.

**Table 2:** Symptoms on admission.

	ORAL group (n = 24)	NG group (n = 24)	P value
Abdominal pain, n ( %)	24 (100)	24 (100)	1.000
Nausea, n ( %)	5 (20.8)	5 (20.8)	1.000
Vomit, n ( %)	11 (45.8)	16 (66.7)	0.146
Fever, n ( %)	8 (33.3)	5 (20.8)	0.330
Diarrhea, n ( %)	1 (4.2)	1 (4.2)	1.000
Jaundice, n ( %)	0 (0)	1 (4.2)	0.312

**Table 3:** Comparison in etiology.

	ORAL group (n = 24)	NG group (n = 24)	P value
Anatomic anomalies	11	9	1.000
Gallstones	0	1	
Infection	4	0	
Hypertriglyceridemia	0	1	
Genetic	0	3	
Idiopathic	9	10	

### Primary outcomes

All participants initiated EN via oral or NG feeding within 48 h after admission. The median duration of abdominal pain in the ORAL group postadmission was 3.0 (1.0 to 4.5) days, whereas the NG group reported a similar median duration of 3.0 (2.0 to 6.0) days (*p* = 0.104). No patient in the ORAL group experienced intolerance; whereas 2 in the NG group did, which required fasting and fluid therapy (*p* = 0.489). MRCP examination revealed that both patients had choledochal cysts, and they subsequently underwent choledochocystectomy. All the patients in the ORAL group successfully tolerated oral feeding. There was no difference in weight changes between the 2 cohorts at discharge, 1 week postdischarge and 5 weeks postdischarge. At the final follow-up visit, both groups exhibited a slight increase in weight (Table 4).

**Table 4:** Outcomes.

	ORAL group (n = 24)	NG group (n = 24)	P value
Time to pain free after admission, d, median (IQR)	3.0 (1.0 to 4.5)	3.0 (2.0 to 6.0)	0.104
Feeding tolerance, n ( %)	24 (100)	22 (91.6)	0.489
Δ in weight at discharge, kg, median (IQR)	0 (−0.8 to 0)	−0.05 (−0.4 to 0)	0.658
Δ in weight at first follow-up point, kg, median (IQR)	0 (−1.0 to 0.5)	0 (−0.3 to 0.7)	0.502
Δ in weight at second follow-up point, kg, median (IQR)	0.8 (−0.6 to 1.2)	0.5 (0 to 1.3)	0.927
Hb on discharge, g/L, median (IQR)	127.0 (124.5 to 131.5)	131.0 (122.0 to 139.0)	0.204
Albumin on discharge, g/L, median (IQR)	43.2 (40.5 to 44.5)	38.5 (37.1 to 41.5)	0.724
PAB on discharge, g/L, median (IQR)	202.0 (174.6 to 233.0)	220.2 (181.5 to 255.0)	0.465
Recurrent abdominal pain, n ( %)	1 (4.2)	0 (0)	1.000
Δ in amylase levels on day 3 of feeding, U/L, median (IQR)	−303.1 (−691.2 to −64.9)	−541.6 (−877.7 to −257.5)	0.130
Δ in lipase levels on day 3 of feeding, U/L, median (IQR)	−208.2 (−364.2 to −25.8)	−853.5 (−2340.8 to −184.8)	0.019*
Δ in amylase levels on discharge, U/L, median (IQR)	−440.7 (−819.4 to −103.2)	−498.9 (−936.6 to −153.7)	0.482
Δ in lipase levels on discharge, U/L, median (IQR)	−208.0 (−740.8 to −21.4)	−464.2 (−1336.1 to −136.6)	0.176
Readmission of recurrent pancreatitis, n ( %)	0 (0)	2 (8.3)	0.489
Complications, n ( %)	0 (0)	4 (16.7)	0.109

Δ difference value. *, P<0.05.

### Secondary outcomes

**Fig. 2:**
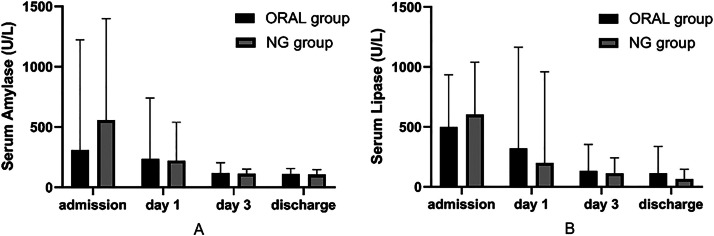
Changes of serum amylase and lipase on different time points. Day 1, the first day of EN. Day 3, the third of EN.

In the ORAL group, one patient experienced recurrent abdominal pain, which improved following a reduction in feeding. The child subsequently showed increased tolerance for further feeding with a gradual increase in quantity. No instances of recurrent abdominal pain were noted in the NG group (*p* = 1.000). Upon discharge, no significant differences in nutritional parameters, including hemoglobin, albumin, and prealbumin levels, were observed between the two groups (Table 4). With respect to the changes in pancreatic enzyme levels, a significant median reduction in lipase levels was observed in the NG group compared with the ORAL group on day 3 of feeding (*p* = 0.019). Additionally, during the oral/NG feeding period and at the time of discharge, no significant differences were observed in the serum amylase and lipase levels or their changes between the two groups of children (Table 4, Figure 2). In terms of complications, there were 4 patients in the NG group, with 3 patients presenting with fever and abdominal pain accompanied by diarrhea, and 1 patient with unexpected tube removal; in contrast, the ORAL group had no cases of complications (*p* = 0.109, Table 4). The first 3 patients were clinically diagnosed with gastroenteritis; however, no viral infection was detected.

Patients in both groups underwent US at baseline and at two follow-up time points. Compliance with the scheduled scans was 100 % in the ORAL group and 87.5 % in the NG group (*p* = 0.234). At the 1-week follow-up, mild pancreatic changes (e.g., swelling, abnormal echogenicity) were observed in 5 children (20.8 %) in the ORAL group, compared with 3 children (14.2 %) in the NG group (*p* = 0.705). At the 5-week follow-up, all children in the ORAL group exhibited normal findings, while 1 child (4.7 %) in the NG group still presented with focally increased echogenicity (*p* = 0.467). Throughout the follow-up period, US assessments revealed no local pancreatic complications in either cohort of patients.

In the NG group, two patients were readmitted to the hospital within 5 weeks post-discharge, whereas no patients in the ORAL group experienced readmission. The first patient presented with 2 heterozygous mutations in SPINK1, whereas the second patient exhibited incomplete pancreatic division; both patients were readmitted due to recurrent pancreatitis despite complete resolution of abdominal pain and normal amylase levels at discharge.

## Discussion

Although guidelines emphasize the value of early oral feeding in pediatric AP, supporting evidence from pediatric RCTs remains limited. Therefore, the authors conducted an RCT specifically in children, comparing oral with NG tube feeding. The present trial demonstrated no significant differences between the two strategies in length of hospital stay, feeding tolerance, abdominal pain duration, readmission rates, or complications in pediatric mild-to-moderate AP. Notably, weight-gain trends did not differ significantly between groups, corroborating earlier findings [15]. Hence, based on the present findings, the authors propose that oral feeding should be the preferred nutritional strategy for children with mild AP to moderately SAP, which aligns with the established adult data [11].

Although patients with AP are often advised to follow a low-fat diet, accumulating evidence questions its necessity. Studies indicated full-fat diets are safe during early EN in children with AP [15], and animal models suggested the inflamed pancreas may be refractory to further stimulation [16]. Consistently, a retrospective analysis found that average dietary fat intake did not significantly affect hospital stay duration or lipase levels in mild pediatric AP [9]. In the present study, employing a polymeric formula (35.5 % fat) and advancing to a full-fat oral diet after reaching nutritional targets yielded favorable recovery outcomes without strict low-fat restriction (< 30 % of calories from fat) [17], lending further support to the safety of full-fat diets in this population.

The EN (oral, gastric, or jejunal) can maintain the integrity of the intestinal mucosa, safeguard against the decline in intestinal immune function, and restricts bacterial translocation [18,19]. NG feeding has been shown to be well tolerated in both adult and pediatric populations with AP [10,14,20], and serves as a suitable nutritional strategy for patients with severe illness, mostly in the ICU or with distinctly decreased oral feeding [2]. All of these participants initiated EN within 48 h, yet two patients in the NG group exhibited symptoms of intolerance. Further examination revealed that they had choledochal cysts and underwent choledochocystectomy. The previous study on tube feeding revealed similar findings [10], which suggested a possible association between choledochal cysts and feeding intolerance; however, further validation is needed.

The literature indicates that prevalent methods of EN access, including nasoenteric tubes, gastrostomy, and jejunostomy, carry risks of mechanical, gastrointestinal, infectious, and metabolic complications [21,22]. The earlier studies also revealed the possibility of accidental tube removal [10]. In a large cohort of 925 critically ill patients with SAP, any form of EN was associated with infections, while oral feeding correlated with the lowest infection and mortality rates [23]. It is therefore understandable that the complication profiles in the present study differed.

Although a serum lipase level greater than 7 times the upper limit of normal within 24 h post-onset could be a suggested predictor for pediatric SAP [24], and although lipase levels were elevated on admission in 2 cohorts, no patient in the present study developed SAP. The inflammatory process in AP is characterized by intra-pancreatic enzyme activation and failure of cellular protective mechanisms [1]. Given the potential for recurrence and atypical presentation, serial monitoring of lipase and amylase was conducted during follow-up, alongside pancreatic ultrasonography and clinical assessments.

The present study has several limitations. First, the etiologies of pediatric AP are varied and distinct from those in adults, as are the severity and overall prognosis associated with pediatric AP [25]. The predominant etiological factors in both cohorts were anatomical anomalies and idiopathic origins, which were similar to the findings of a previous study [25]. Owing to the experimental design, cases resulting from trauma, systemic diseases and pharmacological agents, as well as patients with pre-existing comorbidities, were excluded; therefore, it remains uncertain whether the findings can be generalized to other types of pancreatitis. Second, the authors recruited as many eligible participants as possible, but the sample size was limited (71 % of the intended sample size). Furthermore, given the high risk of life-threatening complications, this study does not address the implementation of early nutritional therapy for SAP and may not be able to comprehensively reflect all AP cases. Additionally, the present study focused on differences in feeding patterns; thus, caloric intake was increased every day until the target amount was achieved. However, more details of feeding, such as how to advance feeding and how much increase in calories and fat, still need to be explored.

## Conclusions

Pediatric AP not only impacts the physical health of children but also imposes additional burdens on their families. Improving treatment strategies for this condition holds long-term significance for both individuals and society. The present research demonstrated that early oral feeding can effectively replace NG feeding in children with mild-to-moderate AP, thereby providing critical support for early nutritional recommendations to minimize unnecessary invasive procedures in young patients. Moving forward, it remains essential to continue in-depth research on early nutritional strategies for severe pediatric AP or AP caused by different causes, and to conduct similar multicenter studies, as the potential impact could be even more profound.

## Contribution of each author

YH, HZ were involved in conceptualization, data curation, formal analysis and writing-original draft. LCF, XYJ, XD were involved in data curation, formal analysis and visualization. LQZ was involved in software and visualization. JDY and JC were involved in methodology, resources and supervision. JC and JGL were involved in project administration, validation and writing-review & editing. All authors agree to be accountable for all aspects of the work.

## Funding

There is no sponsorship/funding in the preparation of this article. All project funding was obtained through internal self-funding sources.

## Clinical trial registration

The trial was registered at the Chinese Clinical Trial Registry (https://www.chictr.org.cn, ChiCTR2100050915).

## Data availability

Deidentified individual participant data (including data dictionaries) will be made available, in addition to study protocols, the statistical analysis plan, and the informed consent form. The data will be made available upon publication to researchers who provide a methodologically sound proposal for use in achieving the goals of the approved proposal. Proposals should be submitted to jingan@zju.edu.cn.

## Conflicts of interest

The authors declare no conflicts of interest.

## Appendix Supplementary materials

Image, application 1
