# Supplementary material for: The utilization of oral feeding in pediatric pancreatitis: a randomized controlled study
Source: J Pediatr (Rio J). 2026 Feb 6;102(2):101501. doi: 10.1016/j.jped.2026.101501 (PMC12907086; doi:10.1016/j.jped.2026.101501)
Supplement: Supplementary file 1 [file mmc1.docx]

**JPED-D-25-00473_ Supplementary Material**

**Supplementary Table 1** Formula composition and caloric breakdown (per 100 grams).

| Components | Energy (kilocalories) |
| --- | --- |
| Fat | 164.7 |
| Protein | 55.6 |
| carbohydrates | 240.0 |
| Other | 3.4 |
| Total | 463.7 |
